# Supplementary material for: Prevalence and correlates of tetanus toxoid uptake among women in sub-Saharan Africa: Multilevel analysis of demographic and health survey data
Source: PLoS One. 2023 Dec 27;18(12):e0296174. doi: 10.1371/journal.pone.0296174 (PMC10752541; doi:10.1371/journal.pone.0296174)
Supplement: Attachment — Submitted filename: Response to Reviewers Comments.docx [file pone.0296174.s002.docx]

Dear Editor,

Authors are pleased to resubmit the revised manuscript for consideration, after a review by reviewers. We believe we have addressed the comments thoroughly.

I present below a point-by-point of the concerns raised and how they have been addressed in this revised submission. The authors revised feedback appears in track changes and appears as the authors' response.

We look forward to your positive feedback on our submission.

Thank you

(Corresponding author)

**Reviewers’ comments**

Reviewer #1: This is a timely and insightful piece of work on the prevalence and correlates of tetanus toxoid vaccination in sub-Saharan Africa. It is clear, concise and outlines in detail the exact variables it set out to measure as well as the specific tests carried out. It also provides important findings that health policy makers can use in interventions. There are however a few corrections and suggestions for you to consider in making the paper more robust.

Response: Thank you for making time to review our manuscript.

1. The variables: ‘health insurance coverage, antenatal care attendance, getting medical help for self: permission to go, getting medical help for self: distance to the health facility, and getting medical help for self: getting money for treatment’ could be added to the contextual variables instead of individual variables. They appear to be the context of the dependent variable.

Response: Thank you. These variables pertain to issues specific to the respondents (women) and are not contextual. Plethora of studies using the DHS dataset also confirms the inclusion of these variables at the individual level and not the contextual level.

2. The variable ‘sex of household head’ is listed as a contextual variable but was not included in the chi-square and multi-level analysis. Is there a reason why?

Response: This variable was dropped and not utilised throughout the study. Hence, its absence from the Tables.

3. While the literature on tetanus immunization uptake have been highlighted, I suggest that you could make use of a theoretical background or framework which provides a logical relationship between the variables and also help establish apriori expectations. The framework would provide a solid rationale for the work. For instance the socio-ecological model can be useful. It will enable you look at the multiple levels of influence on health behaviors or variables and their outcomes. This can also help you delineate the individual level variables and contextual variables properly

Response: We have provided a theoretical model that informed the selection of the variables included in the study.

4. Lastly, the paper will benefit from some proof reading. For instance in the discussion section ‘update’ should read ‘uptake’ and ‘them themselves’ should read ‘ themselves’ .

Response: Thank you. We have thoroughly proofread the manuscript to correct grammatical and typographical errors.
